# Sepsis modulates the human hematopoietic stem cell compartment in peripheral blood and bone marrow

**DOI:** 10.1186/cc14036

**Published:** 2014-12-03

**Authors:** T Skirecki, U Zielińska-Borkowska, J Kawiak, G Hoser

**Affiliations:** 1 Laboratory of Flow Cytometry, The Center of Postgraduate Medical Education, Warsaw, Poland; 2 Department of Anesthesiology and Intensive Care, The Center of Postgraduate Medical Education, Warsaw, Poland

## Introduction

Efficient fight with infection requires robust production of immunocompetent cells. This response is called emergency hematopoiesis and depends on the proliferation of progenitor cells and awakening dormant hematopoietic stem cells (HSCs) into cycling. As during sepsis an altered immune system is often observed, it seems important to reveal the impact of this syndrome on HSCs. Recent discoveries have shown that HSCs circulate in the peripheral blood and may boost local immune response via paracrine mechanisms and differentiation into myeloid cells. Altogether, these rationales led us to investigate the circulating HSCs in septic patients and in the bone marrow (BM) of septic 'humanized mice' transplanted earlier with human HSCs.

## Methods

Samples of peripheral blood were collected from 23 patients with sepsis (on days 1 and 3) and 20 healthy volunteers. The following antigens were analyzed by flow cytometry: CD34, CD38, Ki-67, CD133, Lin and CD45. In order to investigate HSCs in their microenvironment, a model of cecum ligation and puncture (CLP) was performed on the NOD.Cg-Prkdc/scidIL2rγ mice that were transplanted with human cord blood CD34^+ ^cells 8 weeks earlier. BM cells were analyzed 24 hours after CLP by colony-forming unit assay with medium supporting growth of human cells.

## Results

Septic patients had a significantly increased (threefold, *P *< 0.01) number of CD34^+^CD38^- ^HSCs on the third day of the disease. Also, the CD133^+ ^HSC number was increased in septic patients, while CD34^+^CD45^+^Lin^- ^progenitors were detected at much lower level than in controls. Interestingly, Ki-67^+^CD34^+^Lin^- ^cells were fourfold higher in septic patients. Patients with higher number of CD133^+ ^HSCs had significantly lower likelihood of 60-day survival (*P *< 0.05). Analysis of human HSCs from BM of septic mice revealed significantly compromised hematopoietic colonies output (248 vs. 125 in sham animals). CLP caused also expansion of CD34^+^CD38^- ^HSCs in BM and absolute increase of Ki-67^+^CD34^+^Lin^- ^cells (1.5-fold).

## Conclusion

In this work we have observed significant changes in circulating HSCs during sepsis. During the disease, dormant HSCs enter the cell cycle (measured by Ki-67 expression) and are mobilized to the peripheral blood. However, the progenitor cells disappear from circulation. Novel use of humanized mice confirmed expansion of early human HSCs in BM during the sepsis model. Despite expansion of the HSC pool, the amount of functional progenitors in BM is decreased in this model. We suggest that HSCs play a significant role in the course of sepsis and may become a new prognostic and therapeutic target.

